# Usage Patterns of Anticoagulant Therapy in Patients with Mild Cognitive Impairment or Alzheimer’s Disease

**DOI:** 10.1002/alz.086110

**Published:** 2025-01-09

**Authors:** Haixin Zhang, Quanwu Zhang, Amir Abbas Tahami Monfared, Michael C. Irizarry

**Affiliations:** ^1^ Alzheimer’s Disease and Brain Health, Eisai Inc., Nutley, NJ USA; ^2^ Alzheimer’s Disease Franchise, Eisai Inc., Nutley, NJ USA; ^3^ Alzheimer’s Disease & Brain Health, Eisai Inc., Nutley, NJ USA; ^4^ Eisai Inc., Woodcliff Lake, NJ USA

## Abstract

**Background:**

This study aims to describe usage patterns and risk factors associated with anticoagulant therapy in patients with mild cognitive impairment (MCI) or Alzheimer’s disease (AD).

**Methods:**

The United States Medicare claims database (2008– 18) was used to identify patients aged ≥65 years with MCI or AD and to evaluate their anticoagulant use from 2016– 17. A random sample of new anticoagulant users (n = 21,069) was selected. Baseline clinical characteristics during the 12 months prior to therapy initiation were constructed using Charlson Comorbidity Index components. Stable anticoagulant therapy was defined as continued use for ≥4 weeks. Using a nested case‐control design, a control group that did not receive anticoagulants was selected from the MCI/AD sample and matched to those receiving anticoagulants by year of therapy initiation, age, and sex. A mixed‐effects model evaluated the likelihood of stable anticoagulant use with census region as a random effect.

**Results:**

Among 5,379,863 patients with MCI/AD, 487,745 were on anticoagulants (96% stable use). The mean age was 83.6 years (62% women, 86.4% White, 8.4% Black, 3.5% Hispanic, 1.1% Asian, and 1.6% Other/Unknown). The most frequently observed comorbidities were hypertension (86.7%), hyperlipidemia (70.5%), and mental disorder (45%) among those receiving anticoagulants, compared to 74%, 60%, and 44%, respectively, among matched controls. Use of anticoagulants was more likely to be observed in patients with MCI vs AD (odds ratio (OR) = 1.2, *P*<0.05), arrhythmia (OR = 3.41, *P*<0.05), congestive heart failure (OR = 2.04, *P*<0.05), depression (OR = 1.86, *P*<0.05), metastatic cancer (OR = 1.52, *P*<0.05), hypertension (OR = 1.31, *P*<0.05), cerebrovascular disease (OR = 1.31, *P*<0.05), composite indications for therapy such as coronary artery bypass graft/percutaneous coronary intervention, atrial fibrillation, etc. (OR = 1.29, *P*<0.05), and in women vs men (OR = 1.15, *P*<0.05), but less likely with renal disease (OR = 0.8, *P*<0.05). Anticoagulant use increased after 2012 (*P*<0.05). Hispanic patients had the highest rate of anticoagulant use amongst all racial/ethnic groups (OR = 3.1 vs White [*P*<0.05], OR = 1.1 vs Black [*P*<0.05], OR = 1.9 vs Asian [*P*<0.05]).

**Conclusions:**

Patients with MCI were more likely to be stable anticoagulant therapy users than those with AD. Stable use of anticoagulant therapy was highest in Hispanic vs all other racial/ethnic groups, likely due to uncontrolled risk factors of the study.

